# First-Trimester Complete Blood Count as a Predictor of Gestational Diabetes Mellitus: A Multi-center Primary Health Care Study

**DOI:** 10.7759/cureus.89147

**Published:** 2025-07-31

**Authors:** Abdullah M Alzahrani, Noor S Alharbi, Nourah Alageel, Morooj H Alharbi, Dareen A Qattan

**Affiliations:** 1 College of Medicine, King Saud bin Abdulaziz University for Health Sciences, Jeddah, SAU; 2 College of Medicine, King Abdullah International Medical Research Center, Jeddah, SAU; 3 Family Medicine, King Abdulaziz Medical City, Ministry of the National Guard - Health Affairs, Jeddah, SAU; 4 Family Medicine, King Fahd Military Medical Complex, Dhahran, SAU

**Keywords:** biomarkers, cbc: complete blood count, first trimester, gestational diabetes mellitus, inflammation, neutrophil count, ogtt, pregnancy, pregnancy complications, risk assessment

## Abstract

Background

Gestational diabetes mellitus (GDM) is a common pregnancy complication linked to significant maternal and neonatal risks, such as fetal risk of large for gestational age (LGA) and polyhydramnios. This study aimed to evaluate the predictive value of first-trimester complete blood count (CBC) parameters - particularly neutrophil count - for the development of GDM among women attending National Guard primary care centers in Jeddah, Saudi Arabia.

Methods

A retrospective cohort study was conducted using records from January 2023 to January 2024. Pregnant women aged 18-45 years who completed first-trimester laboratory testing and underwent GDM screening at 24-28 weeks were included. Women with pre-existing diabetes, chronic illnesses, or signs of ongoing infections were excluded. Clinical, laboratory, and pregnancy outcome data were analyzed using IBM SPSS Statistics for Windows, Version 29 (released 2022; IBM Corp., Armonk, NY, USA), employing t-tests, chi-square tests, and binary logistic regression to identify significant predictors.

Results

Among 399 women, 25.6% developed GDM. Significant predictors included older maternal age, higher body mass index (BMI), excessive gestational weight gain, and a history of previous GDM (p < 0.001). Laboratory findings showed elevated fasting glucose and red blood cell (RBC) counts in the GDM group, while neutrophil count and neutrophil-to-lymphocyte ratio (NLR) were not statistically significant predictors. Logistic regression confirmed that the one-hour oral glucose tolerance test (OGTT) value, total weight gain, and previous GDM history were independent predictors, with OGTT showing the strongest association (OR = 3.878, p < 0.001).

Conclusion

Traditional risk factors, including maternal age, BMI, weight gain, and GDM history, remain crucial for GDM prediction. First-trimester neutrophil counts did not show predictive value, suggesting limited utility as early inflammatory markers.

## Introduction

Gestational diabetes mellitus (GDM) is a significant global health concern that affects both mothers and their offspring. It refers to any level of glucose intolerance diagnosed for the first time during pregnancy, commonly appearing in the second or third trimester as a result of insulin resistance induced by pregnancy-related hormonal changes. Although GDM often resolves after delivery, it places women at increased risk of developing type 2 diabetes later in life, and carries short- and long-term complications for both mother and baby [1,2].

Globally, the prevalence of GDM has been rising in parallel with increasing obesity and sedentary lifestyles. The International Diabetes Federation reports that around 16.2% of live births are affected by some form of hyperglycemia, with GDM comprising the majority of these cases [2]. In Saudi Arabia, the prevalence is alarmingly high, ranging from 12.3% to 25.5%, depending on the region, driven by factors such as genetics, lifestyle, and urbanization [3,4]. These figures highlight the need for timely screening and risk stratification strategies to reduce maternal and fetal complications.

Complications from unmanaged GDM include preeclampsia, cesarean delivery, macrosomia, shoulder dystocia, birth trauma, neonatal hypoglycemia, and long-term metabolic disorders for the baby [5]. Also, the future risk of type 2 diabetes for the mother is increased once she is diagnosed with GDM. These maternal and fetal risks make early prediction a crucial step. Established risk factors for GDM include advanced maternal age, obesity, ethnicity, family or personal history of GDM, sedentary lifestyle, and poor dietary habits [1,6,7]. Common predictive tools include body mass index (BMI) and fasting plasma glucose (FPG). Recently, interest has grown in identifying novel biomarkers, such as inflammatory markers.

Emerging evidence suggests that systemic inflammation may contribute to GDM pathogenesis. Neutrophils, which play a central role in inflammation, may be implicated in insulin resistance - a hallmark of GDM. Elevated neutrophil counts and an increased neutrophil-to-lymphocyte ratio (NLR) have been observed in women who develop GDM [8,9]. One study found higher neutrophil counts in GDM cases, independently associated with macrosomia and preeclampsia [10]. Another study demonstrated that higher NLR levels were linked to insulin resistance and adverse outcomes [9].

The inflammatory hypothesis proposes that chronic low-grade inflammation - driven by cytokines and reactive oxygen species from neutrophils - disrupts insulin signaling pathways, leading to glucose intolerance [11]. Neutrophils also interact with other immune cells in adipose tissue, amplifying inflammation and contributing to insulin resistance.

Given the potential role of neutrophils in predicting GDM, assessing first-trimester neutrophil count may aid in identifying at-risk women early in pregnancy. This could guide preventive interventions, improve outcomes, and reduce healthcare costs. However, further research is required to confirm these associations and integrate inflammatory biomarkers into existing prediction models [12,13]. Complete blood count (CBC) is one of the first labs routinely ordered in the first trimester for pregnant women. Increased neutrophil count, with different cut-point values, has been noted in recent studies and found to be a significant predictor of GDM. If infection is excluded, it could be a helpful tool and warning sign for early intervention before the development of GDM and its complications. Although such a tool may not be specific, it could be a sensitive and cost-effective way of early risk prediction if confirmed to be significant. The study aimed to assess whether CBC parameters - particularly neutrophil count - predict the development of GDM among women attending National Guard primary care centers in Jeddah.

## Materials and methods

This was a record-based, observational, retrospective cohort study conducted using data from the BestCare system at antenatal care clinics in three National Guard primary health care centers in Jeddah, Saudi Arabia - Alwaha Specialized Polyclinic, Iskan, and Bahrah. The study included data collected between January 2023 and January 2024. All pregnant women aged 18 to 45 who completed laboratory investigations during the first trimester and underwent GDM screening between 24 and 28 weeks’ gestation were included.

Women were excluded if they had pre-existing diabetes, chronic hypertension, morbid obesity (BMI > 40), multiple gestation, chronic comorbidities such as liver or kidney failure, or an infectious illness in the two weeks preceding lab collection. Patients with positive viral serology (e.g., hepatitis B, human immunodeficiency virus (HIV), syphilis) were also excluded.

The calculated minimum sample size was 324, determined using Raosoft software (Raosoft, Inc., Seattle, WA, USA), with a 5% margin of error, 95% confidence level, and 50% response distribution, based on previous estimates from the same healthcare centers. A non-probability convenience sampling method was used to recruit eligible participants from antenatal clinics during the designated period.

Data were retrieved retrospectively from electronic health records using a customized data collection form. The form included demographic and clinical variables such as age, BMI, gravidity, parity, medical history, and laboratory parameters. Laboratory variables included first-trimester CBC with differential (white blood cell (WBC), neutrophils, lymphocytes, monocytes, and platelets), red blood cell (RBC) indices (hemoglobin, mean corpuscular volume (MCV), mean corpuscular hemoglobin (MCH), and mean corpuscular hemoglobin concentration (MCHC)), and first-trimester fasting glucose, compared to oral glucose tolerance test (OGTT) values (fasting, one-hour, and two-hour).

Using IBM SPSS Statistics for Windows, Version 29 (released 2022; IBM Corp., Armonk, NY, USA), descriptive statistics were computed. For continuous variables, the means (M) and standard deviations (SD) were presented, and frequencies with percentages were calculated for categorical variables. To check for statistical significance in differences among women, independent samples t-tests were utilized to compare means of continuous variables between groups. Normality was assumed due to sample size (N > 30 per group), in line with the Central Limit Theorem, but was confirmed through Shapiro-Wilk tests. Chi-square tests of independence were employed for categorical variables to assess differences in proportions. When expected cell counts were minimal (e.g., hypertension during pregnancy with n = 1 and n = 4), Fisher’s exact test was used instead. A binary logistic regression model was used to determine factors that predict GDM. The model calculated regression coefficients (B). Multicollinearity among predictors was verified using variance inflation factors (VIF < 10), with a significant constant (p < 0.001) and predictors (e.g., one-hour OGTT, p < 0.001) indicating a satisfactory fit. Variable selection utilized all anticipated predictors instead of stepwise techniques, as indicated by the thorough output.

The protocol was approved on July 7, 2024, by the Institutional Review Board of the Ministry of National Guard Health Affairs, Jeddah, Saudi Arabia (IRB No. 0000016924). Written informed consent was waived by the IRB, as this study involved retrospective analysis of anonymized data.

## Results

A total of 399 pregnant women were included in the study. Of these, 102 (25.6%) were diagnosed with GDM, while 297 (74.4%) were not. Table 1 describes the clinical features and their significance using the t-test, in which women with GDM were considerably older (M = 33.5 years, SD = 6.12) than those without GDM (M = 30.3 years, SD = 5.54), with a p-value < 0.001. The BMI during pregnancy was significantly higher in the GDM group (M = 28.3, SD = 4.76) compared to the non-GDM group (M = 25.3, SD = 5.01; p < 0.001). Also, weight gain varied significantly between the groups, both before GDM diagnosis (GDM: M = 5.90 kg, SD = 4.39; non-GDM: M = 4.57 kg, SD = 4.14; p < 0.001) and throughout the entire pregnancy (GDM: M = 10.86 kg, SD = 5.67; non-GDM: M = 7.50 kg, SD = 4.65; p < 0.001), indicating a consistent trend of more significant weight gain in the GDM group.

**Table 1:** Clinical characteristics of women with and without GDM. Data are presented as means ± SD, or n (%). *Based on independent samples t-test, **Based on Chi-square, ***Statistically significant (p < 0.05) GDM, Gestational Diabetes Mellitus; BMI, Body Mass Index

Clinical characteristics	GDM		t/x^2^	p-value
	Yes	No		
	N = 102	N = 297		
Age (years)	33.5 ± 6.12	30.3 ± 5.54	4.873*	<0.001***
Pregnancy BMI	28.3 ± 4.76	25.3 ± 5.01	5.009*	<0.001***
Weight gain				
Before GDM diagnosis	5.90 ± 4.39	4.57 ± 4.14	2.651*	<0.001***
Whole pregnancy	10.86 ± 5.67	7.50 ± 4.65	2.728*	<0.001***
Parity				
Nulliparous	21 (20.6%)	96 (32.3%)	5.045**	0.025***
Parous	81 (79.4%)	201 (67.7%)		
Gravidity				
G1-2	31 (30.4%)	151 (50.8%)	12.799**	<0.001***
G3+	71 (69.6%)	146 (49.2%)		
Previous abortion				
Yes	37 (36.3%)	74 (24.9%)	4.878**	0.027***
No	65 (63.7%)	223 (75.1%)		
Previous GDM (n = 282)				
No	46 (56.8%)	180 (89.6%)	38.938**	<0.001***
Yes	35 (43.2%)	21 (10.4%)		
Family history of diabetes mellitus				
Yes	53 (52.0%)	129 (43.4%)	2.225**	0.136
No	49 (48.0%)	168 (56.6%)		
Systolic blood pressure (mmHg)	112.9 ± 11.7	110.8 ± 11.0	1.623*	0.053
Diastolic blood pressure (mmHg)	71.7 ± 8.3	69.8 ± 7.4	2.233*	0.018***

For categorical data, the Chi-square test was used to assess for significant differences. Parity and gravidity displayed remarkable differences. In the GDM group, 79.4% (n = 81) of women had given birth previously, whereas in the non-GDM group, this figure was 67.7% (n = 201; p = 0.025). Likewise, gravidity of G3 or more was more prevalent in the GDM group (69.6%, n = 71), compared to the non-GDM group (49.2%, n = 146; p < 0.001), indicating that having multiple pregnancies elevates the risk of GDM. Moreover, a prior history of abortion was noted in 36.3% (n = 37) of women with GDM, compared to 24.9% (n = 74) of those without (p = 0.027), indicating a statistically significant difference.

For previous GDM history, assessed among 282 parous women, 43.2% (n = 35) of the GDM group reported a prior GDM diagnosis, compared to 10.4% (n = 21) of the non-GDM group (p < 0.001). The family history of diabetes mellitus showed no significant differences between groups, and no significant difference in systolic blood pressure was observed (GDM: M = 112.9 mmHg, SD = 11.7; non-GDM: M = 110.8 mmHg, SD = 11.0; p = 0.053). Nevertheless, the diastolic blood pressure showed a significant difference, with higher readings in the GDM group (GDM: M = 71.7 mmHg, SD = 8.3; non-GDM: M = 69.8 mmHg, SD = 7.4; p = 0.018).

Table 2 shows the laboratory results, in which significant differences using a t-test were observed in multiple parameters. The RBC count was significantly higher in the GDM group (M = 4.41 × 10¹²/L, SD = 0.35) compared to the non-GDM group (M = 4.31 × 10¹²/L, SD = 0.40; p = 0.037). FPG was also higher in women with GDM (M = 4.96 mmol/L, SD = 0.69) than in those without (M = 4.67 mmol/L, SD = 0.48; p < 0.001). Results from the OGTT displayed significant differences after the load: at one hour (GDM: M = 10.28 mmol/L, SD = 1.85; non-GDM: M = 6.96 mmol/L, SD = 1.53; p < 0.001), two hours (GDM: M = 9.24 mmol/L, SD = 2.20; non-GDM: M = 6.24 mmol/L, SD = 1.34; p < 0.001), and three hours (GDM: M = 7.65 mmol/L, SD = 2.28; non-GDM: M = 5.40 mmol/L, SD = 1.01; p < 0.001). Otherwise, there were no statistically significant differences in other laboratory results, including neutrophil count, lymphocyte count, or NLR between groups (p > 0.05).

**Table 2:** Laboratory findings of women with and without GDM. Data are presented as means ± SD. ***Statistically significant (p < 0.05) GDM, Gestational Diabetes Mellitus; WBCs, White Blood Cells; RBCs, Red Blood Cells; Hgb, Hemoglobin; MCV, Mean Corpuscular Volume; MCH, Mean Corpuscular Hemoglobin; MCHC, Mean Corpuscular Hemoglobin Concentration; RDW, Red Cell Distribution Width; OGTT, Oral Glucose Tolerance Test; NLR, Neutrophil-to-Lymphocyte Ratio

Results of laboratory investigations	GDM		t	p-value
	Yes	No		
	N = 102	N = 297		
WBCs (x10^9^/L)	7.32 ± 1.96	7.12 ± 2.13	0.824	0.410
Neutrophil	3.93 ± 1.55	3.90 ± 1.73	0.165	0.865
Lymphocyte	2.53 ± 0.64	2.43 ± 0.74	1.245	0.214
NLR	1.63 ± 0.74	1.75 ± 1.11	1.010	0.313
Platelets	298.35 ± 67.5	294.15 ± 90.53	0.430	0.668
Mean platelet volume	10.06 ± 1.31	10.04 ± 1.36	0.128	0.898
RBCs	4.41 ± 0.35	4.31 ± 0.40	0.150	0.037
Hgb	12.17 ± 0.91	11.95 ± 1.13	1.781	0.076
MCV	85.09 ± 6.98	84.85 ± 9.51	0.235	0.814
MCH	27.74 ± 2.29	27.77 ± 3.35	0.216	0.936
MCHC	32.61 ± 0.90	32.62 ± 1.97	0.230	0.966
RDW	14.28 ± 1.34	14.73 ± 7.41	0.609	0.543
Hematocrit	36.90 ± 4.76	36.54 ± 3.47	0.824	0.410
Fasting plasma glucose	4.96 ± 0.69	4.67 ± 0.48	3.717	<0.001***
OGTT (fasting)	4.94 ± 0.90	4.66 ± 3.09	0.859	0.391
OGTT (1 hour)	10.28 ± 1.85	6.96 ± 1.53	17.879	<0.001***
OGTT (2 hours)	9.24 ± 2.20	6.24 ± 1.34	13.372	<0.001***
OGTT (3 hours)	7.65 ± 2.28	5.40 ± 1.01	4.993	<0.001***

Table 3 describes the pregnancy outcomes for the studied women, in which the Chi-square test was used in most cases, with Fisher’s exact test applied when the expected cell count was minimal (n = 1 to n = 4). The most notable outcome was a significant difference in fetal sex distribution (p = 0.003), showing a higher percentage of female infants in the GDM group (60.8%, n = 62) compared to the non-GDM group (43.9%, n = 130), alongside a correspondingly lower percentage of male infants (GDM: 39.2%, n = 40; non-GDM: 56.1%, n = 166).

**Table 3:** Outcome of pregnancy of women with and without GDM. Data are presented as means ± SD. *Based on independent samples t-test, **Based on Chi Square, ***Statistically significant (p < 0.05) GDM, Gestational Diabetes Mellitus

Outcome of pregnancy	GDM		t/x^2^	p-value
	Yes	No		
	N = 102	N = 297		
Mode of delivery				
Cesarean section	32 (31.4%)	90 (30.3%)	0.840**	0.840
Vaginal delivery	70 (68.6%)	207 (69.7%)		
Postpartum hemorrhage				
Yes	3 (2.9%)	15 (5.1%)	0.376**	0.281
No	99 (97.1%)	282 (94.9%)		
Hypertension during pregnancy				
Yes	1 (1.0%)	4 (1.3%)	0.621**	0.621
No	101 (99.0%)	293 (98.7%)		
Fetal sex				
Male	40 (39.2%)	166 (56.1%)	8.642**	0.003***
Female	62 (60.8%)	130 (43.9%)		
Birth weight	3.02 ± 0.41	3.05 ± 0.46	0.686*	0.493
Birth height	51.03 ± 2.71	50.86 ± 2.63	0.640*	0.646
Head circumference	33.80 ± 1.39	33.58 ± 1.51	1.010*	0.313
Macrosomia				
Yes	0 (0.0%)	8 (2.7%)	0.094**	0.094
No	102 (100.0%)	289 (97.3%)		

Conversely, other pregnancy outcomes exhibited no significant differences. The mode of delivery was similar, with cesarean sections occurring in 31.4% (n = 32) of GDM cases and 30.3% (n = 90) of non-GDM cases, while vaginal deliveries occurred in 68.6% (n = 70) and 69.7% (n = 207), respectively (p = 0.840). Postpartum hemorrhage occurred in 2.9% (n = 3) of the GDM group and 5.1% (n = 15) of the non-GDM group (p = 0.281), while the incidence of hypertension during pregnancy was low in both groups (GDM: 1.0%, n = 1; non-GDM: 1.3%, n = 4; p = 0.621). Neonatal indicators showed no significant differences: birth weight (GDM: M = 3.02 kg, SD = 0.41; non-GDM: M = 3.05 kg, SD = 0.46; p = 0.493), birth height (GDM: M = 51.03 cm, SD = 2.71; non-GDM: M = 50.86 cm, SD = 2.63; p = 0.646), and head circumference (GDM: M = 33.80 cm, SD = 1.39; non-GDM: M = 33.58 cm, SD = 1.51; p = 0.313) were comparable across groups. Macrosomia did not occur in the GDM group (0.0%, n = 0) and was present in 2.7% (n = 8) of the non-GDM group; however, this difference was not statistically significant (p = 0.094).

Logistic regression analysis

Table 4 displays the findings of a logistic regression analysis that determines factors associated with GDM. The model incorporated age, BMI, parity, gravidity, history of previous GDM, one-hour OGTT outcome, and weight gain, using nulliparous parity and a gravidity of 1 as the reference categories.

**Table 4:** Logistic regression for the factors predicting GDM. *Based on independent samples t-test GDM, Gestational Diabetes Mellitus; OGTT, Oral Glucose Tolerance Test; BMI, Body Mass Index

Factors	B		SE	Wald	df	Sig.	Exp(B)	95% CI for Exp(B)	
								Lower	Upper
Age		-0.016	0.039	0.172	1	0.678	0.984	0.911	1.063
BMI		0.073	0.039	3.434	1	0.064	1.076	0.996	1.162
Parity (nulliparous ref)		1.039	0.658	2.489	1	0.115	2.826	0.777	10.270
Gravidity (gravida 1-2 ref)		-0.788	0.644	1.495	1	0.221	0.455	0.129	1.608
Previous history of GDM		1.049	0.503	4.346	1	0.037*	2.854	1.065	7.650
OGTT 1 hr		1.355	0.158	73.634	1	<0.001	3.878	2.846	5.286
Lymphocyte		0.006	0.070	0.007	1	0.934	1.006	0.877	1.153
Neutrophil		-0.199	0.162	1.517	1	0.218	0.819	0.597	1.125
Weight gain whole pregnancy		0.197	0.042	21.756	1	<0.001	1.218	1.121	1.323
Weight gain until GDM test		-0.098	0.052	3.570	1	0.059	0.906	0.818	1.004
Constant		-13.896	2.051	45.894	1	<0.001	0	-	-

Binary logistic regression identified three independent predictors of GDM. The strongest predictor was the one-hour OGTT outcome, which had a notably significant coefficient (B = 1.355, SE = 0.158, p < 0.001). The odds ratio (Exp(B) = 3.878, 95% CI: 2.846-5.286) suggests that, for every 1 mmol/L rise in one-hour OGTT glucose, the likelihood of GDM nearly increases fourfold, highlighting its significant diagnostic importance. Total weight gain throughout pregnancy demonstrated a significant correlation (B = 0.197, SE = 0.042, p < 0.001), with an odds ratio of 1.218 (95% CI: 1.121-1.323). A past history of GDM was another important predictor (B = 1.049, SE = 0.503, p = 0.037), showing an odds ratio of 2.854 (95% CI: 1.065-7.650), which suggests that women previously diagnosed with GDM had almost three times greater odds of recurrence. Conversely, other variables such as neutrophil and lymphocyte counts were not significant predictors in the model.

## Discussion

This study investigated the clinical, laboratory, and pregnancy outcome differences between women with and without GDM, and evaluated predictors of GDM in a primary care setting. The prevalence of GDM in our study cohort was 25.6%, higher than the 19.6% reported in a 2017 study from the same healthcare setting [14]. This rise may reflect increasing obesity rates, sedentary behavior, and dietary shifts in the region, warranting further investigation.

Established clinical risk factors, such as advanced maternal age, higher BMI, and a previous history of GDM, were significantly associated with GDM in our population. Although maternal age did not remain statistically significant in the logistic regression model, its trend aligned with established literature, suggesting reduced insulin sensitivity with aging [15]. Gestational weight gain, both before diagnosis and throughout pregnancy, was also significantly higher in the GDM group and was independently associated with GDM in the regression analysis, supporting previous evidence that excessive gestational weight gain contributes to insulin resistance and glucose intolerance [16].

Obstetric history - specifically, gravidity and parity - was significantly different between groups. Women with GDM were more likely to be multiparous and have a higher number of pregnancies. Prior studies have similarly noted higher GDM risk among multiparous women, likely due to sustained metabolic strain and possible residual β-cell dysfunction from previous pregnancies [5].

A prior history of GDM was a strong and independent predictor in our regression model, with nearly a threefold increase in odds, underscoring the recurrent nature of the condition. This finding emphasizes the importance of monitoring high-risk women closely in subsequent pregnancies [5,15].

Among first-trimester laboratory findings, FPG was significantly higher in women with GDM. This supports earlier findings that linked elevated early pregnancy glucose levels with adverse pregnancy outcomes, such as large-for-gestational-age (LGA) infants and increased cesarean delivery rates. A 2019 study found that a fasting glucose cutoff of 4.5 mmol/L offered moderate sensitivity and specificity in predicting GDM, further emphasizing the importance of early glycemic assessment [17]. Also, RBC count was interestingly found to be significantly elevated in the GDM group, though its clinical interpretation warrants caution. While RBC elevation may reflect mild hemoconcentration or a response to subclinical inflammation, its isolated predictive value could be a highlighted point for future research. This aligns with previous research suggesting hematological shifts in GDM patients, but without consistent clinical significance [12].

While inflammation is hypothesized to play a role in GDM pathogenesis, this study found no statistically significant differences in neutrophil counts or NLR between groups, contradicting some previous findings [8-10]. This may be due to timing, sample characteristics, or confounding variables. Therefore, first-trimester neutrophil count alone may have limited value as a predictive biomarker.

From a neonatal perspective, interestingly, a higher proportion of female infants was observed among GDM cases, although the clinical relevance of this finding is unclear. However, there were no significant differences in delivery mode, anthropometric measurements, or well-established GDM complications such as postpartum hemorrhage or hypertension. This could possibly reflect effective glycemic control and routine antenatal monitoring in our setting, where a multidisciplinary team consisting of an obstetric physician, endocrinologist, diabetes educator, and dietitian is involved once GDM is diagnosed.

The strength of this study is the comprehensive assessment of both clinical and laboratory predictors, allowing for a holistic analysis of GDM risk factors. Although the use of real-world data, rigorous statistical methods, and clear clinical endpoints enhances the study’s validity, some limitations must be acknowledged. These include its retrospective nature, potential selection bias due to convenience sampling, and the lack of data on dietary and physical activity patterns. Also, the lack of longitudinal follow-up data restricts the evaluation of postpartum outcomes or progression to type 2 diabetes.

## Conclusions

In this retrospective cohort study, we found that traditional risk factors - such as maternal age, BMI, total gestational weight gain, and a prior history of GDM - remain significant predictors for the development of GDM. While inflammatory markers, such as neutrophil count and NLR, have been proposed as early predictors, our findings do not support their routine use in first-trimester screening. The one-hour OGTT value stood out as the strongest independent predictor. These results reinforce the value of established clinical tools and highlight the need for future prospective studies with more diverse inflammatory profiles, after ruling out infection within a more specified time period, that may clarify the potential role of immune markers in GDM prediction.
